# Ferroptosis Holds Novel Promise in Treatment of Cancer Mediated by Non-coding RNAs

**DOI:** 10.3389/fcell.2021.686906

**Published:** 2021-06-21

**Authors:** Yuan Zhi, Ling Gao, Baisheng Wang, Wenhao Ren, Kristina Xiao Liang, Keqian Zhi

**Affiliations:** ^1^Department of Oral and Maxillofacial Surgery, The Second Xiangya Hospital, Central South University, Changsha, China; ^2^Xiangya School of Stomatology, Central South University, Changsha, China; ^3^Department of Oral and Maxillofacial Surgery, The Affiliated Hospital of Qingdao University, Qingdao, China; ^4^Key Lab of Oral Clinical Medicine, The Affiliated Hospital of Qingdao University, Qingdao, China; ^5^Department of Oral and Maxillofacial Surgery, Xiangya Stomatological Hospital and School of Stomatology, Central South University, Changsha, China; ^6^Neuro-SysMed, Center of Excellence for Clinical Research in Neurological Diseases, Haukeland University Hospital, Bergen, Norway; ^7^Department of Clinical Medicine (K1), University of Bergen, Bergen, Norway

**Keywords:** ferroptosis, lipid peroxidation, miRNAs, circRNAs, multiple drug resistance, cancer therapy

## Abstract

Ferroptosis is a newly identified form of regulated cell death that is associated with iron metabolism and oxidative stress. As a physiological mechanism, ferroptosis selectively removes cancer cells by regulating the expression of vital chemical molecules. Current findings on regulation of ferroptosis have largely focused on the function of non-coding RNAs (ncRNAs), especially microRNAs (miRNAs), in mediating ferroptotic cell death, while the sponging effect of circular RNAs (circRNAs) has not been widely studied. In this review, we discuss the molecular regulation of ferroptosis and highlight the value of circRNAs in controlling ferroptosis and carcinogenesis. Herein, we deliberate future role of this emerging form of regulated cell death in cancer therapeutics and predict the progression and prognosis of oncogenesis in future clinical therapy.

## Introduction

Cell death is an irreversible mechanism that is associated with metabolism of the internal environment within the eukaryon in order to maintain cellular homeostasis and development in mammals. Recently, ferroptosis has been identified as a type of non-apoptotic cell death (Dixon et al., 2012), leading to a loss of cells without caspase activity and receptor-interacting protein 1 (RIPK1) kinase activity (Yagoda et al., 2007; Yang and Stockwell, 2008). Different from other types of cell death at the biochemical, morphological, and genetic levels, ferroptosis is characterized by an absence of apoptosis, its dependence on iron, and its ability to disrupt intracellular redox balance (Zuo et al., 2020). Once initiated, ferroptosis causes the cell cytoplasm to become round and detached and causes damage to the mitochondria, including condensed mitochondrial membrane densities, reduced or vanished mitochondria crista, and rupturing of the outer mitochondrial membrane (Xie et al., 2016). Meanwhile, ferroptosis is able to reach a dynamic equilibrium in cell metabolism under physiological conditions. On the other hand, ferroptosis presents itself across many diseases, including heart disease, brain damage, and Alzheimer disease when the equilibrium is broken (Stockwell et al., 2017). Additionally, ferroptosis has been highlighted as a powerful weapon against cancers according to an increasing number of studies. Therapies that rely on ferroptosis have also been applied to the clinic, as several cancers are particularly vulnerable to ferroptotic inducers (e.g., erastin, RSL3) and chemotherapeutic agents (e.g., sulfasalazine, sorafenib). Therefore, it is necessary to determine this newly discovered type of cell death, as well as its connection to cancer.

Non-coding RNAs (ncRNAs) used to be considered junk molecules, but have recently incite considerable interest and new insights (Palazzo and Lee, 2015) due to their essential characteristics in gene expression and translational regulation (Nigita et al., 2019). MiRNAs (miRNAs) and long non-coding RNAs (lncRNAs), the two major members of ncRNAs, have vital roles in gene expression and physiological processes (Asma et al., 2019; Javid et al., 2021). It has been reported that similar regulatory methods of miRNAs and lncRNAs also exist in ferroptosis modulation (Mou et al., 2019). As a special ncRNA with a unique structure, circular RNAs (circRNAs) have been proven to control the regulatory processes of ncRNAs as either miRNA sponges or by direct inhibition (Hansen et al., 2013; Memczak et al., 2013). However, the knowledge of the rare and controversial circRNAs in regulating ferroptosis among diverse pathological conditions has not yet been well elucidated. Herein, it is of high value to highlight the characteristics, functions, and mechanisms of circRNAs in different cancer cells. We also provide an overview of recent discoveries of the role of circRNAs in mediating ferroptosis and future application of circRNAs as novel therapeutic targets.

## Definition and Physiological Effects of Ferroptosis

Ferroptosis refers to a novel form of non-apoptotic cell death. The hallmarks of ferroptosis progression include lipid peroxidation, iron dependence, and inhibition of GPX4-dependent antioxidative systems, leading to a lethal accumulation of lipid reactive oxygen species (ROS), which eventually leads to cell death. On the contrary, iron chelators, antioxidative enzymes, and depletion of polyunsaturated fatty acids (PUFAs) prevents cells from ferroptosis. Additionally, accumulation of lethal lipid ROS has a significant function in numerous diseases, including tumorigenesis, neurological diseases, ischemia reperfusion injury (IRI), renal failure, and hematological system diseases. Emerging evidence has demonstrated that the accumulation of iron in pathological regions is a symptom of specific degenerative diseases (Ward et al., 2014). Moreover, previous studies have shown the anti-oncogenic potential of ferroptosis in neoplastic diseases, whereas ferroptosis can eliminate malignant cells with insufficient essential nutrients (Stockwell et al., 2017). Whether ferroptosis can contribute to the pathogenesis of other diseases is not yet clear, though it has been suggested that ferroptosis is a physiological process that widely occurs in mammals, rather than a pathological or organ-specific process. Therefore, future investigations need to be conducted to distinguish the triggers of ferroptotic cell death under physiological or pathological conditions.

## Biochemical Regulation of Ferroptosis

**FIGURE 1:**
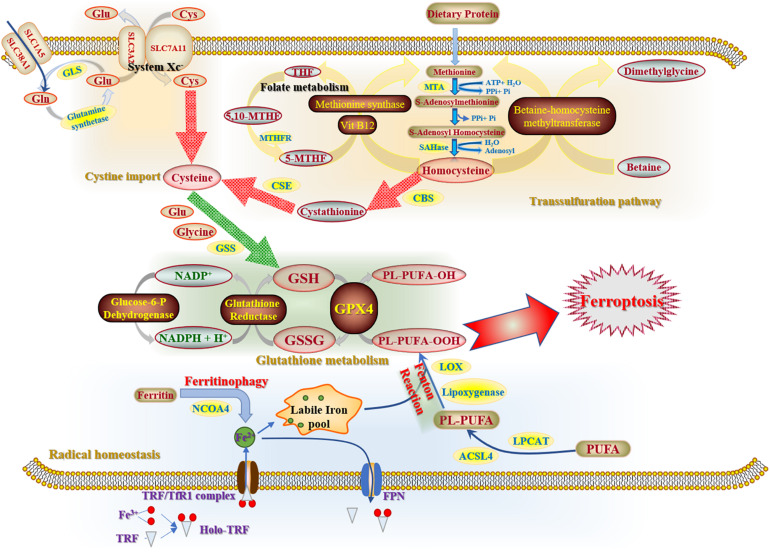
Three main components of ferroptosis mechanisms. Cysteine metabolism, consisting of cystine import and transsulfutation pathway, refers to a compensation method of cellular cysteine that contributes to the biofunction of glutathione (GSH) cycle in cytoplasm. As the key factor of antioxidative system, glutathione metabolism removes excessive ROS with the participation of GPX4 and NADPH. Radical homeostasis is the essential link to exacerbate cell destroy. Labile iron pool is accumulated excessively as a substrate of Fenton reaction for ROS generation by the uptake of ferrous iron and degradation of ferritin.

The initiation and execution of ferroptosis relies on the intersection of amino acid, lipid and iron metabolism (Yagoda et al., 2007). In other words, ferroptosis sensitivity is controlled by several metabolic and biochemical pathways and processes, including cysteine metabolism, glutathione metabolism and radical homeostasis (Figure 1).

### Cysteine Metabolism

Cystine is a disulfide congener of cysteine. Cystine import is driven by system Xc- (Liu N. et al., 2020) with glutamate (Glu) export. As for cellular cysteine, there are two main pathways for cysteine compensation. One pathway is the transmembrane import, which is a rate-limiting process of cysteine accumulation (Bannai and Tateishi, 1986) that is indispensable for glutathione (GSH) biosynthesis and GPX4 bioactivity (Goji et al., 2017). The other is transsulfuration pathway, which acts as a pivotal method to supplement cellular cysteine. Methionine, generated from decomposition of dietary proteins through folate metabolism (Robinson et al., 2018), is an important substrate of transsulfuration pathway and is exerted to synthesize homocysteine catalyzed by ATP-dependent methionine denosyltransferase-induced reaction. In conclusion, these two pathways have jointly ensured the stability of cellular cysteine metabolism and further maintain GSH homeostasis, which is involved in the antioxidative efficacy of radical scavenging.

### Glutathione Metabolism

Glutamine (Gln), a non-essential amino acid, is the most abundant amino acid within the circulatory system of human body. Sufficient cellular Gln is crucial for compensation of GSH, which further enhances the scavenging efficacy to attenuate cellular damage caused by ferroptosis. GPX4 is an essential enzyme that enhances the ferroptosis resistance by transforming lipid hydroperoxides (L-OOH) into the alcohol forms (L-OH), and further maintain cellular redox equilibrium. Besides, nuclear factor erythroid 2-related factor 2 (Nrf2), another pivotal enzyme involved in the cellular antioxidative system, has a positive effect on GSH metabolism by upregulating the radical-scavenging enzyme γ-glutamyl cysteine synthetase (γ-GCS) (Kobayashi and Yamamoto, 2006) and mediating levels of glutathione reductase (GSR). Hence, there is a significant correlation between Nrf2 and GSH metabolism in order to set up resistance to lipid peroxidation (Dayalan et al., 2015).

### Radical Homeostasis

Radical homeostasis is reliant on both iron and ROS metabolism. In iron metabolism, once Fe (II) has combined with transferrin, the transferrin receptor 1 (TfR1), a transmembrane glycoprotein, will internalize this complex and release it into the cytoplasm. The transferrin-bound iron will be stored into a non-toxic form inside ferritins. The process of ferritin’s sequestration and degradation is termed as *ferritinophagy*, during which the labile iron pool can be enriched by degrading mitochondrial heme and ferritins (Mancias et al., 2014). Ferroportin is the only transmembrane protein that is able to export cellular iron (Drakesmith et al., 2015). ROS, a product of lipid peroxidation through the Fenton Reaction, is regarded as a lethal oxidizing agent of cellular oxidative stress. Additionally, polyunsaturated fatty acids (PUFAs) also have an impact on the process of redox equilibrium and sensitivity of cell death. It has been reported that the Ca^2+^-independent phospholipase A_2_β (iPLA2β) can attenuate ferroptosis by hydrolyzing cellular peroxidized phospholipids (Sun et al., 2021).

## Ferroptosis, Potential Value in Neoplastic Diseases

The sensitivity of neoplastic diseases to ferroptosis is highly valued for underlying therapeutic strategies. Ferroptosis may serve as an inhibitory factor for carcinogenesis as neoplastic cells are vulnerable to increased levels of Fe (II) and cellular lipid peroxides (Stockwell et al., 2017). Conversely, due to the upregulation of ferroportin, decreased levels of cellular Fe (II) is a potential defense of cancer cells against ferroptosis to establish a favorable tumor microenvironment for colonization and proliferation (Geng et al., 2018; Bao et al., 2020). Therefore, we propose that ferroptosis may be exerted as a novel approach of tumor suppression due to its inhibition against cell proliferation, tumor migration and invasion.

Classically, inducers of ferroptosis are able to modulate the progression of cell death either directly or indirectly. Erastin is able to selectively inhibit System Xc- antitransport, and decrease cystine uptake in several tumor cell lines (e.g., HT-1080, Calu-1, A-673, Panc-1) (Choi, 1988; Murphy et al., 1989; Zuo et al., 2020). In addition, Ferroptosis-inducing agents 56 (FIN56) can promote GPX4 degradation and exacerbate lipid peroxidation in cancer cells (Shimada et al., 2016). FINO2 can cause suppression of GPX4 activation indirectly, which leads to the execution of ferroptosis (Gaschler et al., 2018). Besides, the direct initiatory agents Ras-selective lethal small molecule 3 (*1S,3R*-RSL3), ML162 (DPI7), and ML210 (DPI10) are able to inactivate GPX4 bioactivity and enhance resistance to lipid peroxidation during carcinogenesis (Yang et al., 2014).

To date, apart from erastin and RSL3 as *ferroptosis-inducing agents* (FIN), several conventional chemotherapeutic drugs (Gout et al., 2001) have shown enhanced therapeutic effects related to ferroptosis. The canonical antimalarial drug artemisinin and its derivatives have been demonstrated as having great value in anticancer therapy (Augustin et al., 2020). Chen G.Q. et al. (2020) reported that artemisinin can trigger ferroptosis by increasing cellular free iron Dihydroartemisinin (DHA), a classical artemisinin derivative, has also been shown to have initiatory effect on ferroptosis in both leukemia cells and head and neck squamous carcinoma cells (HNSCC), as reported by recent studies (Lin et al., 2016; Du et al., 2019). Another derivative artesunate also holds the ability to induce ferroptosis in HNSCC cells, and inhibition of Nrf2-ARE pathway can reverse the resistance of HNSCC to artesunate-induced ferroptosis (Roh et al., 2017). In addition, a previous study on sorafenib revealed that it appeared to trigger ferroptosis in a certain concentration (Dixon et al., 2014). Currently, Li et al. (2021) have reported that artesunate synergizing with sorafenib can trigger ferroptosis in hepatocellular carcinoma. Collectively, these findings suggest that some natural products can serve as ferroptosis inducers and reveal novel strategies for anticancer therapies and further figure out the formation of multidrug resistance (MDR).

Notably, there are several tumor-related genes that play a role in manipulating ferroptosis cell death. Certain mutational forms of the tumor suppressor gene *TP53* has been recognized as having multiple functions in tumor suppression recently, particularly with regards to regulation of ferroptotic metabolism. After acetylating three lysines (*K117R*, *K161R*, and *K162R*), the mutant gene *TP53*^3KR^ is able to induce ferroptosis and suppress tumor growth by inhibiting SLC7A11 and activating ALOX12 expression (Chu et al., 2019). In addition, *TP53* modulates cystine uptake by directly inhibiting SLC7A11 using transcriptional Nrf2 proteins, and inhibits Fe (II) accumulation by inactivating the Nrf2-targeted genes *HO-1* and *FTH11* (Shin et al., 2018). Mutations within another conventional oncogene *Ras* can cause activation of *Ras* proteins and ultimately execute cancer progression. Studies on Ras-mitogen-activated protein kinase (MEK) suggests that upregulation of this oncogenic pathway is able to enhance the sensitivity of cancer cells to ferroptosis by exacerbating lipid peroxidation and generating excess ROS (Ma et al., 2016; Guo et al., 2018). Recent evidence has suggested that *KRas* is a negative regulator for *TP53*, which further promotes Nrf2 expression to halt ferroptosis for tumor growth (Yang et al., 2020). In conclusion, ferroptosis has been linked to carcinogenesis, and has significant value in neoplastic diseases. Application of specific small molecules that can target oncogenes and modulate transcriptional factors of ferroptosis need to be considered and further investigated.

## Non-Coding RNAs, a Prospective Point of Oncogene Encoding

Non-coding RNAs (ncRNAs) refer to RNAs that are transcribed that do not encoding proteins. Since small temporal RNAs were initially discovered in *Caenorhabditis elegans* as lineage defective 4 (*lin-4*) and in mammals as lethal 7 (*let-7*), ncRNAs have been shown to participate in regulating gene expression (Johnsson et al., 2014). The ncRNAs form a physiological control mechanism in mammalian regulatory networks, and participate in another canonical network motif during cancer development (Anastasiadou et al., 2018).

Among ncRNAs, microRNAs (miRNAs) are the most intensively studied ncRNAs in the modulation of carcinogenesis. MiRNAs are endogenous small RNAs, between 21 and 24 nucleotides long, and can be transferred from cell-to-cell by several ways (e.g., exosomes) to alter genetic or epigenetic phenotypes of cancer (Seyed et al., 2020; Maryam et al., 2021). MiRNAs function by base-pairing with complementary sequences within mRNA molecules, leading to inhibition of gene expression at the transcriptional level on the basis of miRNA-mRNA base pairing (Rupaimoole and Slack, 2017). In cancer cells (e.g., gynecologic cancer), some miRNAs function as oncogenes, while others function as tumor suppressors (Zahra et al., 2021). Numerous studies have demonstrated that miRNAs act as oncogenes in cancer development (Neda et al., 2021). Importantly, previous reports have revealed that miRNAs may influence the effect of ferroptosis inducers. Exosomal miR-4443 has been reported to inhibit cisplatin-induced ferroptosis and promote cisplatin resistance in NSCLC (Song et al., 2021). In lung adenocarcinoma cell A549, Deng et al. (2021) found that miR-324-3p can enhance cisplatin-induced ferroptosis by targeting GPX4 directly. Moreover, Ma et al. (2021) reported that miR-424-5p knockdown sensitized ovarian cancer cells to erastin and RSL3 and executed ferroptosis by targeting ACSL4. These findings suggest that miRNAs may have a key regulatory function in MDR, thereby holding great promise for the development of novel and effective therapies for cancer treatment.

With the explanation of miRNA efficacy in cancer treatment, more attention has been paid to ncRNAs in order to investigate whether other ncRNAs, such as circRNAs, have similar bioactivity as miRNAs in order to modulate the carcinogenic processes. Moreover, several reports have assessed the potential effectiveness of circRNAs to repress the miRNA-mediated mRNA editing via absorbing tumorigenic regulators as miRNA-sponges (Hansen et al., 2013; Memczak et al., 2013; Li et al., 2015; Wang et al., 2016; Zheng et al., 2016; Peng et al., 2017). Notably, with a specific circular structure stabilized by covalent bonding, circRNAs suggest a steady regulatory potency of oncogenesis in cytoplasm of cancer cells.

Taken together, based on current biofunctions of ncRNAs in cancer growth, this review has focused on exerting these molecular regulators in ferroptosis and pay greater attention to the promising efficacy of circRNAs, which has not been reported until now.

## Role of ncRNAs in Ferroptosis and Its Molecular Pathways

Recently, ncRNAs have become increasingly recognized as playing a significant role in mediating the development of cancer (Slack and Chinnaiyan, 2019). LncRNAs and miRNAs are the two most widely studied ncRNAs. MiRNAs and lncRNAs, both of which can act alone or interact with each other, take part in regulating the inter-related steps and genetic mediators of programmed cell death, including ferroptosis.

Notably, miRNAs are significantly involved in regulating ferroptosis among cancer cells (Bridges et al., 2012). In melanoma cells, miR-9 is able to inhibit catalytic efficacy of glutamic-oxaloacetic transaminase 1 (GOT1) and halt transamination of a-ketoglutarate (a-KG), while inhibition of miR-9 causes accumulation of lipid ROS, and eventually exacerbates the enforcement of ferroptosis (Zhang et al., 2018). In addition, upregulation of miR-137 causes inhibition of glutamine transporter SLC1A5, and protects cellular proliferation and colonization against ferroptosis in melanoma (Luo et al., 2018). MiR-7-5p, which has an effect on cell migration and invasion in melanoma (Giles et al., 2013), is associated with radio-resistance of cancer cells by decreasing expression of ferrous iron within the cytoplasm (Tomita et al., 2019). Studies in melanoma also suggest specific targets of ferroptosis in order to further identify modulatory efficacy of miRNAs.

GSH homeostasis is known to be a key factor of oncogenesis. Recent studies have highlighted the function of specific miRNAs in modulating intracellular GSH levels. For example, miR-4715-3p is inhibited by overexpression of AURKA (aurora kinase A), a serine threonine kinase that has a significant role in mitotic progression in both normal cells and cancers (Li et al., 2018). Reconstitution of miR-4715-3p has been validated to be an essential method that hinders the process of tumorigenesis by inhibiting GPX4 and enhancing cisplatin sensitivity (Gomaa et al., 2019). Other miRNAs, including miR-185, can prevent human colon adenocarcinoma cells from sustaining oxidative damage by upregulating glutathione peroxidase 2 (GPX2) (Maciel-Dominguez et al., 2013). In gastric cancer, miR-103a-3p directly represses GLS2 expression, which helps maintain cellular Gln levels and prevents execution of ferroptosis (Niu et al., 2019). Moreover, several other miRNAs, including miR-155 (Chen et al., 2017), miR-144-3p (Sun et al., 2017), miR-28 (Yang et al., 2011), miR-181-c (Jung et al., 2017), miR-93 (Singh et al., 2013), and miR-142 (Wang N. et al., 2017), have been identified to downregulate Nrf2 and promote ferroptosis in cancer cells. Taken together, several molecular targets of glutathione metabolism can be specific transcriptional targets for miRNAs, which are known to be tightly associated with the ferroptosis and oncogenesis.

As mentioned previously, SLC7A11 is an important factor that protects cells against ferroptosis due to the antioxidative efficacy enhanced by cystine uptake for cellular GSH metabolism. In oral squamous cell carcinoma (OSCC), miR-375 acts as a tumor suppressor by inhibiting SLC7A11 expression, leading to ferroptosis (Wu et al., 2017). Similar regulatory pathways have been identified for miR-26b in breast cancer (Liu et al., 2011). Thus, it is clear that certain miRNAs execute ferroptosis in cancer cells by inhibiting SLC7A11 expression (Table 1).

**TABLE 1:** Biofunction of miRNAs in regulating ferroptosis in cancer cells.

miRNA	Target gene	Expression changes	Biofunction	Cancer types	Ferroptosis progression	Model	Cell lines	References
miR-9	GOT1	Downregulation	Inhibit lipid peroxidation and iron accumulation	Melanoma	Suppression	Cell culture	A375, G-361	Zhang et al., 2018
miR-9-5p	GOT1	Downregulation	Inhibit glutamine metabolism and redox homeostasis	Pancreatic cancer	Suppression	Cell culture	H6c7, BxPC3, Panc1, Miapaca2, AsPC1, CFPAC1	Wang J. et al., 2019
miR-133b	GST-π	Downregulation	Modulate intracellular glutathione metabolism	Ovarian cancer	Promotion	Cell culture	A2780, A2780/Taxol, A2780/DDP, OVCAR3	Chen et al., 2015
miR-103a-3p	GLS2	Downregulation	Inhibit transportation of glutamate Modulate glutamine metabolism	Gastric cancer	Suppression	Cell culture	MGC-803, MKN-415	Niu et al., 2019
miR-122	GLS2 SLC1A5	Downregulation	Modulate glutamine metabolism	HCC	Promotion	Mice model	EC4	Sengupta et al., 2020
miR-137	SLC1A5	Downregulation	Decrease glutamine uptake and MDA accumulation; Increase sensitivity ferroptosis	Melanoma	Suppression	Cell culture	A375, G-361	Luo et al., 2018
miR-375	SLC7A11	Downregulation	Modulate cystine metabolism	OSCC	Promotion	Cell culture	Hs680, Fadu, SCC-25, CAL-27, Tca8113	Wu et al., 2017
miR-27a	SLC7A11	Downregulation	Mediate regulation of intracellular glutathione	Bladder cancer	Promotion	Cell culture	Ej/T24, RT112	Drayton et al., 2014
miR-4715-3p	AURKA	Downregulation	Inhibit GPX4 bioactivity Enhance cisplatin sensitivity	Gastrointestinal cancer	Promotion	Cell culture	OE33, MKN45, STKM2	Gomaa et al., 2019
miR-522	ALOX15	Upregulation	Inhibit lipid-ROS accumulation	Gastric cancer	Suppression	Male nude mice	SGG-7901, MGC-803 MKN-45	Zhang H. et al., 2020
miR-214-3p	ATF4	Downregulation	Enhance erastin-induced lipid peroxidation	Hepatoma	Promotion	Nude mice	HepG2, Hep3B	Bai et al., 2020
miR-17-92	A20/ACSL4	Downregulation	Inhibit lipid peroxidation	Endothelial cells	Suppression	Cell culture	HUVEC	Xiao et al., 2019
miR-205	ACSL4	Downregulation	Modulate abnormal lipid metabolism	HCC	Promotion	Cell culture	HepG2	Cui et al., 2014
miR-144	Nrf2	Downregulation	Suppress Nrf2/HO-1 pathway Reverse chemoresistance to 5-FU	HCC	Promotion	Cell culture	Bel-7402, Bel-7402/5-FU	Zhou et al., 2016
miR-28	Nrf2	Downregulation	Mediate Nrf2 protein degradation in Keap1-independent manner	Breast cancer	Promotion	Cell culture	MCF-7, HEK293T	Yang et al., 2011
miR-432	Keap1	Downregulation	Upregulate NRF2 protein Enhance NRF2 stabilization	ESCC	Suppression	Cell culture	HeLa, SH-SY5Y	Akdemir et al., 2017
miR-148b	ERMP1/Nrf2/HIF-1	Downregulation	Increase intracellular ROS level	Endometrial cancer	Promotion	Cell culture	RL95-2	Qu et al., 2018
miR-507	Nrf2/ME1	Downregulation	Inhibit Nrf2-mediated oncogenic pathway	ESCC	Promotion	Cell culture	HeLa, LK-2, A549, JHH-5	Yamamoto et al., 2014
miR-155	Nrf2	Upregulation	Decrease intracellular ROS level	Lung cancer	Suppression	Cell culture	A549	Gu et al., 2017
miR-340	Nrf2	Downregulation	Inhibit Nrf2/NQO-1/HO-1 expression	HCC	Promotion	Cell culture	HepG2, HepG2/CDDP	Shi et al., 2014
miR-365	Nrf2	Downregulation	Decrease Nrf2 expression; Increase intracellular ROS generation	HCC	Promotion	Cell culture	HepG2	Gao et al., 2018
miR-378	Nrf2	Upregulation	Decrease intracellular ROS generation	NSCLC	Suppression	Cell culture Animal model	NCI-H292, NCI-H460, A549, SK-MES-1	Skrzypek et al., 2013
miR-125b	PRXL2A/Nrf2	Downregulation	Increase intracellular ROS level Suppress Nrf2 expression	OSCC	Promotion	Cell culture	SAS, OECM1, HSC3, FaDu, OC3, 293T, NOK	Chen et al., 2019
miR-29b-1-5p	AKT/Nrf2	Downregulation	Increase intracellular ROS level	Breast cancer	Promotion	Cell culture	MDA-MB-231	De Blasio et al., 2020
miR-141	Keap1	Downregulation	Modulate Keap1 expression Active Nrf2/IKKβ pathway	Ovarian cancer	Suppression	Cell culture	A2780, A2780/DDP	van Jaarsveld et al., 2013
miR-200a	Keap1	Downregulation	Active Keap1/Nrf2 pathway	ESCC	Suppression	Cell culture	KYSE150, KYSE180, KYSE410, KYSE510	Liu et al., 2015

**FIGURE 2:**
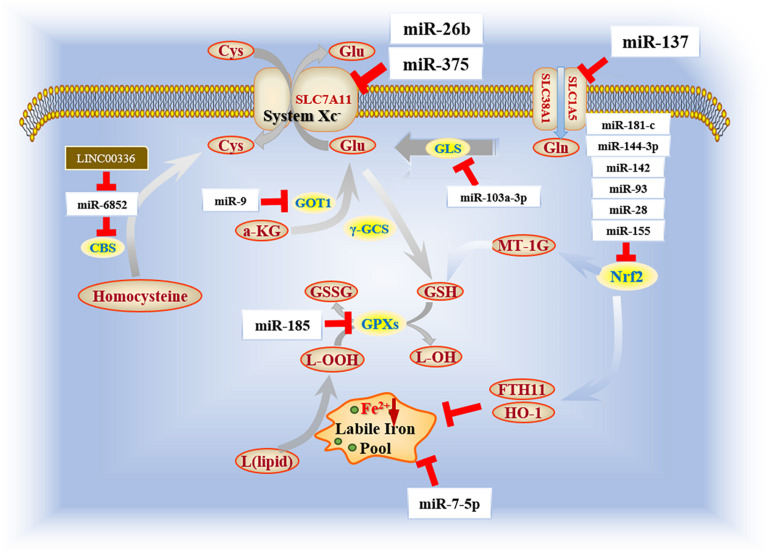
Biofunction of miRNAs in ferroptosis mechanism. Current reports focus on several essential enzymes (e.g., Nrf2, GLS, and GPXs) that impact ferroptosis progression. Among those, Nrf2 holds an irreplaceable role in the regulatory network because of its double value in both ferroptosis and apoptosis.

Recently, scientists have paid significant attention to the relationship between miRNAs and lincRNAs in the regulation of transcription of ferroptosis and oncogenesis. LINC00336, a type of lincRNA with large intergenic transcripts that cover over 200 nt, has been reported to enhance proliferation of lung cancer cells and inhibit ferroptosis through an ELAVL1-dependent manner (Wang M. et al., 2019). Consistent with CBS, miR-6852 can aggravate ferroptosis and restrict tumorigenesis by negatively regulating the bioactivity of CBS, which mediates ferroptosis inhibition. This modulation is protected by overexpression of LSH, which can attenuate p53 recruitment to the promoter region of *ELAVL1*. Studies have revealed a role for LINC00336 as a competing endogenous RNA (ceRNA) to inactivate miR-6852 in lung cancer, thus allowing it to be potential target of lung cancer therapy. To date, limited studies have evaluated the interaction between lincRNAs and ferroptosis. However, studies have shown that, similar to miRNAs, lincRNAs can act as dual regulators for ferroptosis, either by absorbing certain miRNAs to alter their effects or combining with certain enzymes to have an effect on the bioactivity of cancer cells (Figure 2).

Collectively, miRNAs have important regulatory functions, either as tumor suppressors or oncogene inducers, by downregulating transcription of essential genes that are associated with tumorigenesis or ferroptosis or degrading exon generations at the mRNA level. It is plausible that these miRNAs can promote ferroptosis by inactivating certain genes. Therefore, there is an urgent need to explore the relevant molecular mechanisms that underlie miRNAs, as well as to identify the role of miRNAs in the mechanisms of classical anticancer drugs, such as cisplatin and sorafenib.

## circRNA and Ferroptosis

Advanced research approaches on lincRNAs have provided guidance for perspective studies on other lncRNAs, such as circRNAs, which are RNAs that are formed with covalently closed continuous loops. There are three categories of circRNAs, including exonic circRNA (ecircRNA), which are cyclized from exon, circular intronic RNA (ciRNA) from introns and exonic-intronic circRNA (EIcircRNA) (Miri et al., 2012; Memczak et al., 2013). CircRNAs have diverse gene-regulatory functions, and can act as miRNA sponges, protein translators (Legnini et al., 2017), and protein scaffolds. To date, studies have revealed unique characteristics of circRNAs, including abundance, conservation and tissue-specificity, all of which may serve as biomarkers of certain pathological processes, compared to miRNAs (Guo et al., 2014; Wang et al., 2014; Xia et al., 2017).

Previous studies have assessed the functional roles of circRNAs in several physiological and pathological conditions, particularly in the development and diagnosis of cancer (Table 2). Increasing evidence shows that circRNAs mediate autophagy, apoptosis and cell proliferation (Bahn et al., 2015; Du et al., 2017; Gao et al., 2017; Zeng et al., 2017). Most circRNAs are characterized by the high stability and specificity (Salzman et al., 2013). There are several detecting methods (e.g., RT-qPCR, *in situ* hybridization, high-throughput sequencing) that are mature enough for circRNA profiles. CircRNAs are reported to abnormally express in many cancers, such as hepatocellular carcinoma (Aishanjiang et al., 2021), renal cell carcinoma (Frey et al., 2021), glioblastoma (Wei et al., 2021), and gastric cancer (Jiang et al., 2021). Recent studies show that circRNAs are able to reverse resistance to conventional therapeutic strategies in several cancers, such as circLIFR (Zhang et al., 2021b), ciRS-122 (Wang et al., 2020), and Cdr1as (Yuan et al., 2019) in chemoresistance and circATRNL1 (Chen G. et al., 2020) in radioresistance. Taken together, circRNAs are reliable biomarkers in diagnosis, prognosis, and potential targets in clinical therapies.

**TABLE 2:** Our previous studies related to circRNAs in osteogenesis and carcinogenesis.

circRNA	Expression	Target	Biofunction	References
circCDR1as	Up	AKT/ERK_1/2_/mTOR	Promoting autophagy in OSCC cells via sponging miR-671-5p Enhancing OSCC cells viability under a hypoxia microenvironment	Gao et al., 2019a
circPKD2	Down	miR-204-3p/APC2	Upregulating APC2 expression via sponging miR-204-3p Inhibiting OSCC carcinogenesis	Gao et al., 2019b
hsa_circ_0072387	Down	miR-29-3p/miR-141-3p-MMP2/BCL2/PTEN	A potential biomarker in OSCC diagnosis	Dou et al., 2020b
circFLNA	Up	Fucoidan/circFLNA	Inhibiting OSCC progression	Zhang N. et al., 2020
mmu_circ_0001066	Up	miR-16	Restoring the BP-mediated suppression of osteoclasto-genesis via sponging miR-16	Zhang et al., 2021c
circCDK8	Up	mTOR	Repressing the osteogenic differentiation of PDLSC by triggering autophagy activation in a hypoxic microenvironment	Zheng et al., 2021
ciRS-7	Up	miR-7/RAF-1/PIK3CD	Enhancing metastatic progress-ion of OSCC via sponging miR-7	Dou et al., 2020a

**FIGURE 3:**
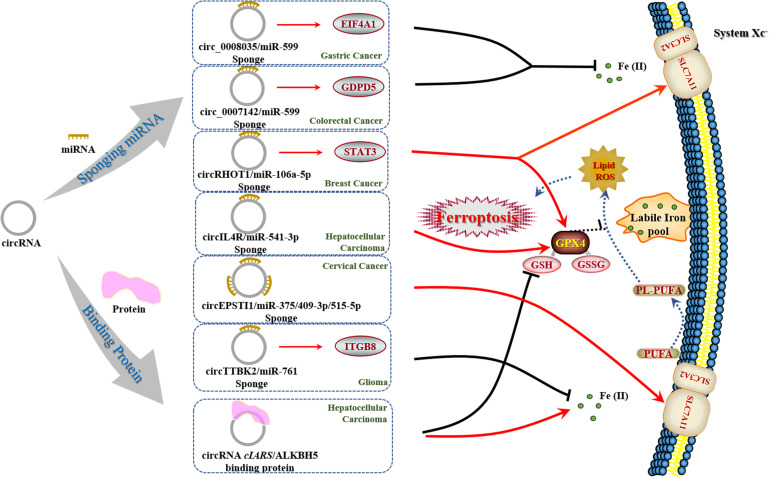
Regulatory effect of circRNAs on ferroptosis in cancers. In the regulation of ferroptosis, circRNAs present two distinct mechanisms based on recent reports. There is only one circRNA (termed *cIARS*) that is able to execute ferroptosis in cancer cells as reported. Moreover, *cIARS* holds novel promise in interacting with proteins, which needs to be deeply addressed for further work.

Currently, only limited reports on the function of circRNAs in ferroptosis have been explored so far (Li et al., 2020; Liu Z. et al., 2020; Xu et al., 2020; Zhang H. Y. et al., 2020; Wang et al., 2021; Wu et al., 2021; Zhang et al., 2021a; Table 3). Most of these studies have focused on the sponging effect of circRNAs, while Liu Z. et al. have brought insights into the role of circRNA *cIARS* as a protein scaffold in mediating RNA binding protein AlkB Homolog 5 (ALKBH5) (Liu Z. et al., 2020). Moreover, they have established a sorafenib-treated HCC model to further explore the mechanism underlying ferroptosis and autophagy. By reviewing these papers, we highlight the multiple mechanisms of circRNAs in regulating ferroptosis (e.g., miRNA sponges, protein scaffolds) (Figure 3). As circRNAs have been reported to regulate the transcription and be translated into proteins (Verduci et al., 2020), we assume that it is of high value for further research to detect the similar mechanisms of circRNAs in regulating ferroptosis and carcinogenesis. Besides, future investigations can establish experimental models as Liu Z. et al. (2020) did to explain the participation of circRNAs and ferroptosis in chemotherapeutic drugs against tumors, which may serve as guidance for potential applications in clinic.

**TABLE 3:** Current reports related to circRNAs in modulating miRNA-regulated ferroptosis progression.

circRNA	Target	Biofunction	Cancer types	Ferroptosis progression	Cell lines	References
circ-0008035	miR-599/EIF4A1	Inhibiting miR-599	Gastric cancer	Up	GE8-1 HGC-27 AGS	Li et al., 2020
circ-TTBK2	miR-761/ITGB8	Inhibiting miR-761	Glioma	Down	LN229 U251 NHA	Zhang H. Y. et al., 2020
circ-clARS	ALKBH5 (RBP)	Siliencing ALKBH5	HCC	Up	HepG2 SMMC-7721 Huh-7	Liu Z. et al., 2020
circ-IL4R	miR-541-3p/GPX4	Sponging miR-541-3p	HCC	Down	THLE-2 HuH-7 HCCLM3	Xu et al., 2020
circ-EPSTI1	miR-375/miR-409-3p/miR-515-5p-SLC7A11	Sponging miRNAs to upregulate SLC7A11 expression	Cervical cancer	Up	CaSki HeLa HcerEpic	Wu et al., 2021
circ-RHOT1	miR-106a-5p/STAT3	Contributing to malignant progression and attenuating ferroptosis	Breast cancer	Down	MDA-MB-231 T47D	Zhang et al., 2021a
circ-0007142	miR-847-3p/GDPD5	Upregulating GDPD5 and reversing miR-847-3p-mediated tumor inhibition	CRC	Down	HCT116 SW620 SW480	Wang et al., 2021
						

CircRNAs provide a promising value in the treatment of MDR of cancers, particularly when targeting the relationship between circRNAs and ferroptosis. Accordingly, ferroptosis has paved the way to develop gene-oriented strategies and more precisive gene therapies than conventional anticancer drugs. Shin et al. (2018) have discovered that activation of the Nrf2-ARE pathway contributes to the resistance of HNC cells to GPX4 inhibition, and inhibition of this pathway reverses resistance to ferroptosis among HNC cells. MT-1G has been demonstrated as a novel regulator of ferroptosis in HCC cells. The molecular mechanism of MT-1G’s role in sorafenib resistance involves inhibiting ferroptosis. Inhibition of MT-1G by RNA interference increases glutathione depletion and lipid peroxidation (Sun et al., 2016), which demonstrates that CAFs secrete exosomal miR-522 in order to inhibit ferroptosis in cancer cells by targeting ALOX15 and blocking lipid-ROS accumulation, resulting in decreased chemo-sensitivity of cisplatin (Zhang H. et al., 2020). However, further investigation into the role of circRNAs in cancer diagnosis and prognostic estimates needs to be addressed.

Based on previous evidence, we can assume that certain circRNAs likely act as miRNA sponges in order to inhibit modulation of downstream transcription in cancer cells, and guide the probable direction of elucidating the underlying features of circRNAs in classical ferroptosis or tumorigenic mechanisms. Furthermore, circRNAs can exert advantages of molecular construction, which directly affects upstream transcriptions. CircRNAs are able to be translated into proteins, which suggests that applications of their expression as regulators can disturb upstream genes. Nevertheless, many controversial views have emerged in circRNAs research and application. As only a limited number of circRNAs have been reported as miRNA sponges, while others only have few miRNA binding sites (Wang et al., 2014), are there other methods that circRNAs take in regulating target mRNAs? Moreover, how can circRNAs protein-translating effect be distinguished from others? Additionally, it is unclear whether circRNAs are degraded or inhibited at the end due to its unique structure. Taking into consideration, circRNAs, which are a controversial type of ncRNAs, likely have distinctive mechanisms in interacting with other ncRNAs and modulating certain cellular processes, such as ferroptosis.

In brief, there is a considerable value of bioactivity among circRNAs which can contribute to further studies in ferroptosis progression and oncogenic regulation, despite the fact that the relationship between ferroptosis and circRNAs requires more identification.

## Discussion

In conclusion, ferroptosis not only helps maintain a dynamic equilibrium under physiologic conditions, but also acts as an effective biomechanism to hinder tumorigenic progression in cancer therapies. The efficacy of tumor suppression is executed by dysregulating cystine uptake, glutathione metabolism and redox homeostasis. Mediating ferroptosis may help provide specific molecular targets for suppressing oncogenesis, and can be used in estimating prognosis of neoplastic diseases. As more ferroptosis inducers have been identified, several compounds have emerged as key factors to protect organisms from malignant tumors by exacerbating cell death at the translational level. Moreover, studies of *erastin* indicate that these ferroptosis inducers likely have multiple targets in regulating ferroptosis mechanisms and influence the expression of ncRNAs, such as miRNAs. Importantly, with the exception of the canonical anticancer drugs, several non-neoplastic drugs have been identified as exerting their capacity to promote the ferroptosis process and transcriptional regulation in cancer cells. Intriguingly, artemisinin and its derivatives have shown enhanced anticancer values synergizing with classical antitumor drugs. These findings contribute to identifying viable pharmacological therapies in cancer treatment. Thus, ferroptosis is likely regarded as an effective clinical strategy in order to avoid chemo-resistance in anticancer treatment.

Numerous studies have recognized miRNAs as being able to modulate oncogenes and genes involved in ferroptosis. miRNAs provide potential gene therapy of cancer treatment by intentionally promoting exacerbation of ferroptosis and dysfunction of oncogenic expression at the transcriptional level. It has been reported that the interaction between miRNAs and anticancer drugs has diverse effects on oncogenesis. Some miRNAs (e.g., miR-522, miR-4443) are able to enhance resistance to cisplatin by inhibiting ferroptosis (Zhang H. et al., 2020; Song et al., 2021), while others (e.g., miR-4715-3p, miR-324-3p) sensitize cancer cells to cisplatin via induction of ferroptosis (Gomaa et al., 2019; Deng et al., 2021). In addition, the bioactivity of circRNAs has been implicated in the progression of ferroptosis and oncogenic regulation. With a unique structure, circRNAs are more stable and specific in expression compared to miRNAs. It has been widely reported that circRNAs prevalently exist in cancers and serve as diagnostic biomarkers of diverse tumors (Wang Y. et al., 2017; Zhong et al., 2018). CircRNAs are able to not only disrupt regulation of miRNAs in circRNA-miRNA-mRNA regulatory networks, but also interacting with transcripts, proteins and even translating proteins to target oncogenes. CircRNAs have a promising future in an underlying pharmacological efficacy that is involved in investigating MDR in chemotherapeutic cancer strategy. Using the concrete MDR experiments, such as constructing antidrug models, the chemotherapeutic value of ferroptosis will become more practical for pharmacological explorations. Further research on specific enzymes of ferroptosis that circRNAs mediate in cancer cells needs to be addressed in the future.

In summary, although regulatory ncRNA, especially circRNAs, are complex and interactions with ferroptosis will require considerable work to unravel, circRNAs can be considered dependable diagnostic and therapeutic molecular biomarkers for cancer in manipulation of cell death. They also facilitate the development of chemoresistance to anticancer drugs and provide a genetic approach for better diagnosis, predicting prognosis and treatment response to cancer.

## Author Contributions

KQZ and YZ had the idea for the article. BSW, LG, and WHR collected the references, performed the literature search, and data analysis. YZ and KXL drafted the first manuscript and prepared the figures and tables. KQZ and KXL designed and corrected the final manuscript. All authors contributed to the study conception and design and read and approved the final manuscript.

## Conflict of Interest

The authors declare that the research was conducted in the absence of any commercial or financial relationships that could be construed as a potential conflict of interest.

## Abbreviations

a-KG: a-ketoglutarate
AKT: protein kinase B
ALOX12: arachidonate 12-lipoxygenase
ALKBH5: AlkB Homolog 5
ARE: antioxidant responsive element
AURKA: aurora kinase A
CBS: Cystathionine- β-synthase
circRNA: circular RNA
DAT: dihydroartemisinin
ELAVL1: embryonic lethal abnormal vision-like RNA-binding protein
ERK: extracellular regulated protein kinases
ESCC: esophageal squamous cell carcinoma
Fe(II): ferrous iron
FIN: ferroptosis-inducing agents
γ-GCS: glutamyl cysteine synthetase
Gln: glutamine
GLS2: glutaminase 2
Glu: glutamate
GOT1: glutamic-oxaloacetic transaminase 1
GPX2: glutathione peroxidase 2
GPX4: glutathione peroxidase 4
GSH: glutathione
GSR: glutathione reductase
GSSG: glutathione oxidized
IRI: ischemia reperfusion injury
let-7: lethal-7
lin-4: lineage defective 4
lncRNA: long non-coding RNA
MDR: multiple drug resistance
MEK: mitogen-activated protein kinase kinase
miRNA: micro RNA
mTOR: mammalian target of rapamycin
MT-1G: metallothionein 1G
ncRNA: non-coding RNA
Nrf2: nuclear factor erythroid 2-related factor 2
NSCLC: non-small cell lung carcinoma
OSCC: oral squamous cell carcinoma
PDLSC: periodontal ligament stem cells
PIK3CD: phosphatidylinositol-4,5-bisphosphate 3-kinase catalytic subunit delta
PUFA: polyunsaturated fatty acid
RIPK1: receptor interacting protein 1 kinase
ROS: reactive oxygen species
RSL3: Ras-selective lethal small molecule 3
SAT1: spermidine/spermine N1-acetyltransferase1
SLC1A5: solute carrier family 1 member 5
SLC7A11: solute carrier family 7 member 11
SLC38A1: solute carrier family 38 member 1
TfR1: transferrin receptor 1.
